# Interval breast cancer risk associations with breast density, family history and breast tissue aging

**DOI:** 10.1002/ijc.32731

**Published:** 2019-11-12

**Authors:** Tuong L. Nguyen, Shuai Li, Gillian S. Dite, Ye K. Aung, Christopher F. Evans, Ho N. Trinh, Laura Baglietto, Jennifer Stone, Yun‐Mi Song, Joohon Sung, Dallas R. English, Mark A. Jenkins, Pierre‐Antoine Dugué, Roger L. Milne, Melissa C. Southey, Graham G. Giles, Malcolm C. Pike, John L. Hopper

**Affiliations:** ^1^ Centre for Epidemiology and Biostatistics, Melbourne School of Population and Global Health University of Melbourne Parkville VIC Australia; ^2^ Department of Clinical and Experimental Medicine University of Pisa Pisa Italy; ^3^ Centre for Genetic Origins of Health and Disease University of Western Australia Perth WA Australia; ^4^ Department of Family Medicine, Samsung Medical Center Sungkyunkwan University School of Medicine Seoul South Korea; ^5^ Department of Epidemiology School of Public Health Seoul National University Seoul South Korea; ^6^ Institute of Health and Environment Seoul National University Seoul South Korea; ^7^ Cancer Epidemiology Division Cancer Council Victoria Melbourne VIC Australia; ^8^ Precision Medicine, School of Clinical Sciences at Monash Health Monash University Clayton VIC Australia; ^9^ Department of Epidemiology and Biostatistics Memorial Sloan Kettering Cancer Center New York NY

**Keywords:** body mass index, breast density, family history, interval breast cancer, Pike's model of breast tissue aging

## Abstract

Interval breast cancers (those diagnosed between recommended mammography screens) generally have poorer outcomes and are more common among women with dense breasts. We aimed to develop a risk model for interval breast cancer. We conducted a nested case–control study within the Melbourne Collaborative Cohort Study involving 168 interval breast cancer patients and 498 matched control subjects. We measured breast density using the CUMULUS software. We recorded first‐degree family history by questionnaire, measured body mass index (BMI) and calculated age‐adjusted breast tissue aging, a novel measure of exposure to estrogen and progesterone based on the Pike model. We fitted conditional logistic regression to estimate odds ratio (OR) or odds ratio per adjusted standard deviation (OPERA) and calculated the area under the receiver operating characteristic curve (AUC). The stronger risk associations were for unadjusted percent breast density (OPERA = 1.99; AUC = 0.66), more so after adjusting for age and BMI (OPERA = 2.26; AUC = 0.70), and for family history (OR = 2.70; AUC = 0.56). When the latter two factors and their multiplicative interactions with age‐adjusted breast tissue aging (*p* = 0.01 and 0.02, respectively) were fitted, the AUC was 0.73 (95% CI 0.69–0.77), equivalent to a ninefold interquartile risk ratio. In summary, compared with using dense breasts alone, risk discrimination for interval breast cancers could be doubled by instead using breast density, BMI, family history and hormonal exposure. This would also give women with dense breasts, and their physicians, more information about the major consequence of having dense breasts—an increased risk of developing an interval breast cancer.

## Abstract 2

**What's new?**

Breast cancers that are detected between regular screening intervals are more aggressive and have poorer outcomes than screen‐detected tumors but no valid risk model exists to predict “interval” breast cancers. Here the authors combined breast density with other breast cancer risk factors such as body mass index, family history and a novel measure of age‐adjusted hormonal exposure. Applying this to more than 150 women with interval breast cancer, they find that risk discrimination can be doubled with the combination of factors as compared with just using breast density alone, thus improving risk predictions and clinical recommendations for breast screening procedures.

**Abbreviations**
AUC: area under the receiver operating characteristic curve
BIC: Bayesian Information Criterion
BMI: body mass index
CC: craniocaudal
CI: confidence interval.
LL: log‐likelihood
OPERA: odds per adjusted standard deviation

## Introduction

Interval breast cancers are diagnosed after a negative screen but before the next recommended screen. Interval cancers are important for many reasons, not the least because women diagnosed with interval cancers have poor outcomes.^1^ Compared with screen‐detected breast cancers, interval breast cancers are generally larger and have a more aggressive phenotype.^2^ Interval cancers have an incidence of 10–20 per 10,000 women attending two‐yearly mammographic screening^3^ and represent 20–30% of all breast cancers diagnosed in those women.^4^

A risk model for interval cancers would be important for breast cancer control. Women could be triaged according to their risk for tailored screening,^5^ a concept being tested by the Women Informed to Screen Depending On Measures of risk (WISDOM) trial.^6^ Triaging could address the on‐going need to improve the cost‐effectiveness of screening, minimize harms^7^ and help mitigate the problem of dense breasts masking tumors,^8^, ^9^, ^10^ an issue made prominent by the late Nancy Cappello.^11^

Dense breasts have become an important issue in the US with most states passing laws that require women with dense breasts to be notified and encouraged to discuss supplemental screening and concerns with their health care provider. More than 25 million US women aged 40–74 years (43%) have dense breasts defined by the Breast Imaging Reporting and Data System's (BI‐RADS) categories c and d.^12^, ^13^ Breast density notification within the US typically depends solely on BI‐RADS classifications, with no consideration of other risk factors or demographics.^14^ Kerlikowske *et al*.^4^ showed that better prediction of interval breast cancer risk can be achieved by including other information, although in doing so, they assumed that the risk factors for breast cancer, in general, apply to interval cancers with the same risk gradient.

In this article, we define breast density as the proportion of the breast image that is mammographically dense, defined by the light or bright areas on a mammogram. Women with greater breast density are more likely to be diagnosed with interval cancers,^15^, ^16^ likely due to the reduced ability of radiologists to detect tumors in dense regions (masking).^15^, ^17^ Breast density, once adjusted for age and body mass index (BMI) due to negative confounding, is implicated in risk of breast cancer overall.^18^

The association of breast density with interval cancers could be due to, or amplified by, a combination of factors related to risk and masking. For example, having a mother or sister with breast cancer has perhaps an even stronger risk association with interval breast cancers than it does with screen‐detected cancers.^2^, ^15^

To better inform women of the consequences of their breast density, we aimed to develop a risk model for interval breast cancer based on breast density and other breast cancer risk factors by conducting a matched case–control study nested within a prospective cohort. We also introduced a novel measure of hormonal exposure, age‐adjusted breast tissue aging. Pike *et al*.^19^ modeled age‐specific breast cancer incidence as a function of breast tissue aging, which summarized the effects of exposure to estrogen and progesterone on incidence by a single measure based on reproductive and hormonally related factors. Rosner *et al*.^20^ confirmed the model and extended it to take into account having one or more full‐term births (see Table 2 and fig. 4 of Rosner et al.^21^). We also studied these putative risk factors, individually and combined, and fitted interactions to address whether their risk associations are modified by breast density.^21^

## Materials and Methods

### Sample

We used the Melbourne Collaborative Cohort Study of adult residents of Melbourne, Australia aged 40–69 years when recruited between 1990 and 1994.^22^, ^23^, ^24^ We conducted a nested case–control study within the cohort of 20,444 women who were unaffected when they completed their baseline questionnaire and who, from data linkage in 2009, were found to have attended BreastScreen Victoria (BSV) at least once. BSV is part of Australia's national breast cancer screening program, established in 1992 to offer free mammographic screening to women over 40 years of age. The target group for the screening program is women aged 50–69 years, who are invited for screening every 2 years. Incident breast cancers were defined as those diagnosed within 2 years of a negative screening at BSV^16^ and identified by BSV or by linkage with the population‐complete Victorian Cancer Registry or the Australian Cancer Database. Incident breast cancers diagnosed less than 2 years after a negative mammogram taken at BSV were classified as interval cancers. Case patients were women diagnosed with incident interval breast cancers. Control subjects were matched to case patients on the year of birth, year of baseline interview and country of birth.^16^, ^24^ Control subjects were randomly selected from women who had not been diagnosed with breast cancer at the age of diagnosis of the case patient. The study sample was 168 interval breast cancer patients (148 with invasive cancer) and 498 matched control subjects (we had aimed for four control subjects per case patient but this was not always possible). The study was approved by the human research ethics committees of the University of Melbourne and Cancer Council Victoria and consent was obtained from study participants at the time of recruitment.

### Measurement of breast density

All mammograms were screen‐film and were digitized using an Array 2905 Laser Film Digitizer at 12‐bit depth. Breast density was measured from the mammograms taken closest to baseline. For interval breast cancer patients, mammograms of the unaffected (contralateral) breast taken at or before diagnosis were used.

We used the CUMULUS computer‐assisted thresholding method to measure conventionally defined breast density.^25^ Five operators (TLN, YKA, SL, CFE and HNT) independently measured breast density blinded to the case–control status. Measurements were completed in sets of 100 mammograms. For the average of operators, the within‐set repeatability was 0.95 (95% confidence interval [CI] 0.94–0.96) and the between‐set repeatability was 0.95 (95% CI 0.93–0.97).

### Measurement of other risk factors

Subjects completed interviewer‐administered questionnaires at baseline that asked about age at menarche, number of full‐term live births, age at each birth, breastfeeding, menopausal status, age at menopause, hormonal replacement therapy use, oral contraceptive use and alcohol consumption. Height and weight were measured using standard protocols and used to calculate BMI = weight (kg)/height (m)^2^. Family history of breast cancer was defined as having at least one first‐degree blood relative with breast cancer based on a questionnaire filled out by women at their time of screening by BSV.

### Breast tissue aging

After Pike *et al*.^19^ and Rosner *et al*.,^20^ starting at puberty, the rate of breast tissue aging is assumed to be a constant (which, without loss of generality, is taken to be 1) up until first full‐term livebirth, when it decreases. Breast tissue aging decreases with subsequent live births and after menopause. The model parameters were estimated so that the area under the breast tissue aging curve to age *t*, taken to the power of 4.5, fits the age‐specific incidence curve for breast cancer. We therefore defined breast tissue aging to be the area under the breast tissue age curve up to the woman's age at baseline questionnaire; see Supplementary Material.

### Statistical methods

For both breast density and breast tissue aging, a cube‐root function gave the optimal Box–Cox power transformation to normality. Transformed breast density was adjusted for age and BMI, and transformed breast tissue aging was adjusted for age, using linear regression. The standardized adjusted breast density and standardized adjusted breast tissue aging were calculated by estimating the mean of the transformed measures for the controls (only) to establish the population norms. We then used these population norms to derive the residuals, for both the controls and the cases, and then divided these control‐based residuals by their respective standard deviations for the controls.

We used conditional logistic regression to estimate the odds ratio (OR) for a binary factor and, for each continuous variable, the change in odds per standard deviation of that variable after it had been adjusted, as above (OPERA).^26^ Statistical inference was based on asymptotic likelihood theory. We used the likelihood ratio test and the Bayesian information criterion (ΔBIC) to compare model fits. Strength of evidence between two models was interpreted as nonexistent if ΔBIC<2, positive if 2–6, strong if 6–10 and very strong if ≥10.^27^

Risk discrimination was assessed from considering log(OPERA), the risk gradient on the log‐odds scale, which is the difference between case patients and control subjects in the means of their standardized and normalized risk factor adjusted for covariates. The equivalent interquartile risk ratio is approximately OPERA^2.5^.^26^ Risk discrimination was also assessed by the AUC; under normality and multiplicative risk assumptions, AUC = Φ[log(OPERA)/√2], where Φ is the cumulative distribution function of the standard normal distribution^28^(Supplementary Material). All statistical analyses were conducted using the software Stata 14.0.^29^

## Results

Table 1 shows that case patients differed from control subjects in breast density for women with and without a family history (*p* = 0.06 and *p* < 0.0001, respectively). Having at least one first‐degree relative with breast cancer was more common for case patients than control subjects (*p* = 0.00006).

**Table 1:** Study participant characteristics by family history

	No family history			Family history		
	Cases	Controls		Cases	Controls	
	(*n* = 132)	(*n* = 450)		(*n* = 36)	(*n* = 48)	
	Mean (SD)	Mean (SD)	*p*	Mean (SD)	Mean (SD)	*p*
Age at mammogram (years)	53.9 (7.5)	53.8 (7.2)	0.96	54.5 (7.3)	53.8 (9.0)	0.7
Age at menarche (years)	13.2 (1.6)	13.1 (1.6)	0.4	12.6 (1.4)	13.2 (1.4)	0.03
Age at menopausal (years, post, *n* = 401)	48.7 (5.0)	47.3 (6.3)	0.07	48.9 (6.0)	46.8 (6.4)	0.3
Body mass index (kg/m^2^)	26.6 (5.1)	26.5 (4.9)	0.7	26.8 (5.9)	26.6 (5.1)	0.8
Number of live births (*n* = 564)	2.6 (0.9)	2.7 (0.9)	0.4	2.8 (0.9)	2.9 (0.9)	0.9
Age at first live birth (*n* = 564)	26.2 (5.0)	25.5 (4.3)	0.2	25.8 (3.5)	24.3 (4.9)	0.2
Age at last live birth (*n* = 522)	31.4 (4.7)	31.0 (4.7)	0.5	31.5 (3.6)	30.9 (5.4)	0.6
Breast tissue aging	37.9 (4.8)	37.6 (4.8)	0.6	38.8 (4.7)	37.0 (5.7)	0.1
Nulliparous (n = 102)	36.4 (6.5)	37.4 (6.5)	0.6	36.2 (5.4)	36.8 (7.7)	0.9
Parous (n = 564)	38.1 (4.5)	37.7 (4.5)	0.3	39.7 (4.2)	37.1 (4.8)	0.03
Breast density (%)	21.1 (10.9)	14.8 (9.1)	<0.0001	19.1 (9.2)	15.1 (9.6)	0.06

	*n* (%)	*n* (%)	*p*	*n* (%)	*n* (%)	*p*
Parous (yes)	112 (85)	390 (87)	0.6	27 (75)	35 (73)	0.8
Postmenopausal (yes)	80 (61)	273 (61)	0.99	21 (58)	27 (56)	0.8
Breast fed (ever)	102 (77)	355 (79)	0.7	25 (69)	33 (69)	0.9
Alcohol consumption (ever)	89 (67)	285 (63)	0.4	30 (83)	33 (69)	0.1
Hormonal therapy use (ever)	36 (45)	104 (38)	0.3	5 (24)	8 (30)	0.7
Oral contraceptive use (ever)	81 (61)	296 (66)	0.4	21 (58)	30 (63)	0.7

**Figure 1:**
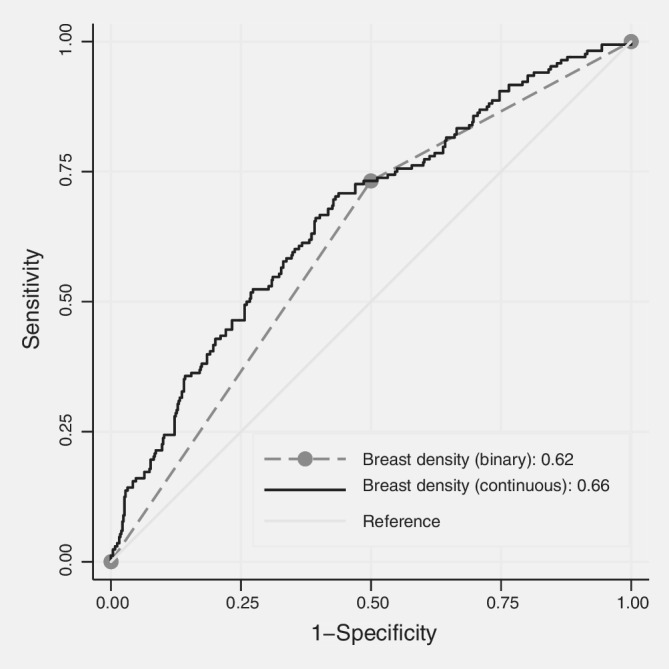
Receiver operating characteristic curves for predicting interval breast cancer using breast density as a binary variable based on the median for controls and using breast density as a continuous variable unadjusted for any covariates.

Figure 1 shows that, for unadjusted breast density, the AUCs were 0.62 and 0.66 (standard errors ~0.02) when dichotomized about the median and when used as a continuum, respectively. (For BI‐RADS as four categories, the AUC was 0.65 with χ^2^ = 43.10). There was very strong evidence that the latter gave an improved fit compared with the two other measures (both ΔBIC≥15). The OR for the upper *versus* lower 50% was 3.2 (95% CI 2.1–4.8). Compared with the lowest quartile, the ORs for the second, third and fourth quartiles were 1.9 (95% CI 1.0–3.8), 3.4 (95% CI 1.8–6.5) and 5.9 (95% CI 3.1–11.2), respectively.

Table 2 shows that the OPERA for transformed and standardized breast density unadjusted for age and BMI was 1.99 (95% CI 1.61–2.46; *p* < 0.0001), and there were no associations with either age at mammogram (a matching variable) or BMI (both *p* > 0.2; analyses not shown). Table 2 also shows that, after adjustment for age and BMI, the OPERA for adjusted breast density was 2.26 (95% CI 1.81–2.81; *p* < 0.0001), and there was very strong evidence (2ΔLL = 16) that this gave a better fit than unadjusted breast density.

**Table 2:** Univariable associations with risk of interval breast cancer

	OPERA (95% CI)	AUC (95% CI)	χ^2^	LL
Breast density^1^	1.99 (1.61–2.46)	0.66 (0.62–0.71)	48.36	−201.90
Age at mammogram	0.66 (0.36–1.19)	0.51 (0.46–0.56)	1.98	−225.09
Body mass index	1.02 (0.85–1.22)	0.50 (0.45–0.55)	0.05	−226.05
Adjusted breast density^2^	2.26 (1.81–2.81)	0.70 (0.65–0.74)	66.93	−192.62

1 Breast density is defined as percent mammographic density.

2 Adjusted for age and body mass index.

Abbreviations: AUC, area under the receiver operating characteristic curve; CI, confidence interval; OPERA, odds per adjusted standard deviation; χ^2^ is the likelihood ratio test statistic.

Table 3 shows that, when fitted together, the association of adjusted breast density with interval breast cancer was unchanged after adjustment for the other factors. The association with family history was also virtually unchanged after adjustment for adjusted breast density.

**Table 3:** Univariable and multivariable associations with risk of interval breast cancer

	Univariable				Multivariable	
Adjusted breast density^1^	2.26 (1.81–2.81)	–	–	–	2.24 (1.79–2.81)	2.24 (1.79–2.81)
Family history	–	2.70 (1.66–4.39)	–	–	2.65 (1.57–4.49)	2.65 (1.56–4.48)
Body mass index	–	–	1.01 (0.97–1.04)	–	–	1.00 (0.97–1.04)
Adjusted breast tissue ageing^2^	–	–	–	1.17 (0.97–1.40)	1.07 (0.87–1.30)	1.07 (0.87–1.31)
AUC	0.70 (0.65–0.74)	0.56 (0.53–0.59)	0.50 (0.45–0.55)	0.54 (0.49–0.59)	0.72 (0.67–0.76)	0.72 (0.67–0.76)
χ^2^	66.93	15.46	0.10	2.79	80.35	80.39
Log likelihood	−192.62	−218.35	−226.03	−224.68	−185.91	−185.89

Note: For binary risk factors the association is an odds ratio (OR) whereas for continuous risk factors, the association is the change in odds ratio per standard deviation of the adjusted risk factor (OPERA). AUC, area under the receiver operating characteristic curve; χ^2^ is the likelihood ratio test statistic.

1 Adjusted for age and body mass index.

2 Adjusted for age.

Table 4 shows that there was marginal or strong evidence that the adjusted breast tissue aging association differed by family history and breast density (*p* = 0.04, 0.07 and 0.003, respectively; see Models 1 and 2).

**Table 4:** Multivariable associations (95% confidence intervals in parentheses) with risk of interval breast cancer, allowing for interactions between adjusted breast density or family history and adjusted breast tissue aging

	Model 1	Model 2	Model 3
Adjusted breast density	2.27 (1.81–2.85)	–	2.36 (1.85–3.00)
Family history	–	2.63 (1.58–4.38)	2.56 (1.47–4.45)
Adjusted breast tissue aging	0.90 (0.72–1.13)	1.09 (0.88–1.33)	0.79 (0.61–1.02)
			
*Interactions*			
Adjusted breast tissue aging × adjusted breast density	1.34 (1.10–1.63)	–	1.38 (1.12–1.70)
Adjusted breast tissue aging × family history	–	1.72 (1.00–2.96)	2.10 (1.17–3.79)
AUC	0.71 (0.66–0.75)	0.58 (0.53–0.63)	0.73 (0.69–0.77)
χ^2^	75.59	22.93	95.01
Log likelihood	−188.29	−214.61	−178.57

Abbreviations: AUC, area under the receiver operating characteristic curve; χ^2^ is the likelihood ratio test statistic.

Table 4 also shows evidence for interactions between adjusted breast tissue aging and both adjusted breast density and family history when all three factors were fitted together (see Model 3). Fitting the two interaction terms improved the fit (*p* < 0.001) and both interaction terms were significant (*p* = 0.001 and 0.007, respectively).

**Figure 2:**
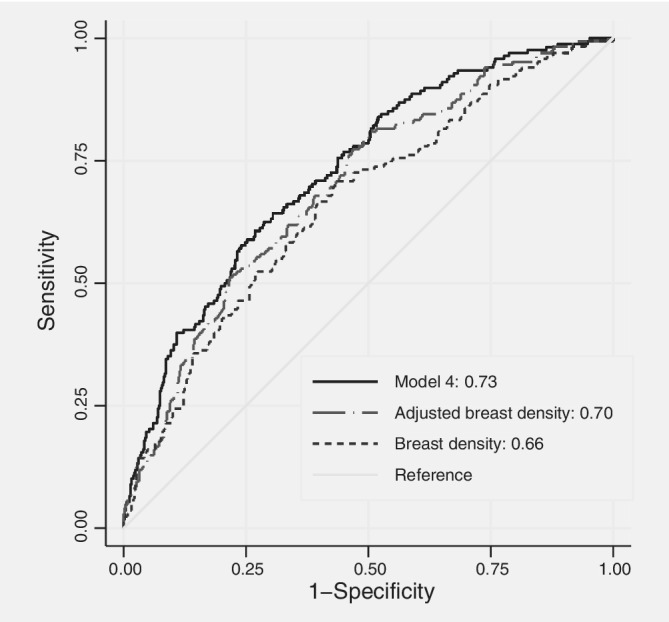
Receiver operating characteristic curves for predicting interval breast cancer using breast density as a continuous variable unadjusted for any covariates, using breast density adjusted for age and body mass index and using the best fitting model that included interactions.

Figure 2 shows that the AUC from fitting adjusted breast density, family history and their interactions with adjusted breast tissue aging was 0.73 (95% CI 0.69–0.77), equivalent to OPERA = 2.34 and a ninefold interquartile risk ratio. This AUC was greater than 0.70 (95% CI 0.65–0.74) when using adjusted breast density alone (*p* = 0.003) and greater than 0.66 (95% CI 0.62–0.71) when using unadjusted breast density as a continuum (*p* = 0.0001).

The AUCs of 0.73, 0.70 and 0.66 above correspond to log(OPERA)s of 0.86, 0.73 and 0.60, respectively, which are equivalent to IQRRs of 9.0, 6.2 and 4.5. Therefore, compared with using only unadjusted breast density, on the log(OPERA) scale the risk gradient increased by 43% from using adjusted breast density as a continuum, including family history, and allowing for interactions between age‐adjusted breast tissue aging and both adjusted breast density and family history. In terms of AUC compared to 0.5, the corresponding increase was from 0.66 to 0.73 (44%). The increase from considering dense breast as a binary construct was from 0.62 to 0.73 (190%).

## Discussion

We found that the ability to predict interval breast cancers might be almost doubled by considering more than unadjusted breast density dichotomized about its median, similar to how dense breasts are defined using BI‐RADS. Interval cancers are either (*i*) potentially detectable at screening but missed (about 20–25% of all interval cancers) or (*ii*) nonexistent, too small, or otherwise not detected at screening but grew rapidly thereafter. Possibility (*i*) is more likely the greater the proportion of the breast covered by light or bright areas.^16^ Possibility (*ii*) is more likely the greater a woman's underlying risk of breast cancer risk, especially that of more aggressive disease.^2^, ^30^ Either way, women more likely to develop breast cancer overall are more likely to develop interval cancers.

First, unadjusted breast density was a better predictor of interval cancer than breast density dichotomized by the median for controls, analogous to the current BI‐RADS classification. Second, breast density was a better predictor of interval cancer when adjusted for age and BMI. Third, having a family history of breast cancer was an important predictor of interval cancers.

Fourth, the risk associations of breast density adjusted for age and BMI and family history combine multiplicatively. This implies that, in terms of absolute risk of interval cancer, family history is more important the greater a woman's breast density. This observation is of relevance to cancer family genetic services, and the role of breast density in predicting interval cancers for women already at increased (familial) risk deserves attention.

Fifth, a novel breast cancer risk factor which combines absolute risk information from age, age at puberty, number and timing of any live births and age at menopause (if achieved) to represent the relative risk due to exposure to estrogen and progesterone for women of the same age,^19^ interacted with breast density and family history. This suggests that, for women of the same age, their hormonal exposure is more important the greater their risk of having a tumor masked and the greater their familial risk. Including information on reproductive and familial risk factors could improve risk prediction, but not necessarily in the same way or to the same degree as it would if it was assumed it had the same risk association with breast cancer overall. In practice, whether or not to include asking women about their reproductive history could depend on whether they had high breast density or a family history.

Our finding of a more than multiplicative combination of breast tissue aging with breast density and family history, is not the only example of such interactions for interval cancers. Li *et al*.^31^ recently found evidence for interactions between breast density and a measure of genetic risk whereby, for women with interval cancer, those with low breast density had a greater chance of carrying a high‐risk breast cancer susceptibility mutation.

Figure 1 shows that moving from dense breasts as a binary variable to breast density as a continuous variable improved sensitivity (true positivity) with little loss in specificity when specificity was high. Figure 2 shows that adding risk factor information improved specificity (true negativity) with little loss in sensitivity when sensitivity was high.

The BI‐RADS classification of dense breast based on a–b *vs*. c–d is similar to our classification based on the median for controls. The increased risk associated with having dense breasts, defined as being in the upper half of the breast density distribution, was about threefold. Given that the incidence of interval breast cancers is about 1–2 per 1,000 women undergoing 2 yearly screening,^3^ the incidence of interval breast cancer for women with dense breasts is about 1–2 per 1,000 higher than it is for women without dense breasts. If a woman has 10 (2 yearly) screens in her lifetime and is always categorized as having dense breasts, her risk of ever having an interval breast cancer will be about 1–2% more than for a woman without dense breasts. Given that >25 million women in the US aged 40–74 years have dense breasts,^13^ dense breasts are implicated in about 25–50,000 interval cancers among these women.

Our model almost doubled the risk gradient and could be used to derive more optimal cut‐offs for management strategies based on risk stratification. This empirical information could be useful for screening programs to conduct economic modeling and cost–benefit analyses. Decisions about how to use the model to inform future screening and use of other screening modalities should be based on absolute risk, not only on relative risk, so a model for the age‐specific incidence of interval cancer needs to be created specifically for the population being screened.

Strengths of our study include the use of a novel risk measure based on Pike's model of breast tissue aging and fitting interactions. We did not presume the same risk factors, with same risk gradients, applied to interval cancers as they do to breast cancer overall and found evidence that contradicted this assumption.

Weaknesses include the relatively small sample size so our risk discrimination is likely to be overestimated and there is a need for independent replication. Our findings were based on film mammograms and might not apply to digital mammograms. They were also based on only one mammogram; considering a woman's breast density history could improve risk prediction. We based our model on information collected at or around the baseline, on average 5–6 years prior to diagnosis,^35^ so it is likely that the risk gradients could be even greater if predictions were over a 2‐year interval after a negative screen. Finally, we only had information for family history as a binary construct and no information on genetic risk.

Risk prediction could also be substantially improved. We recently created Cirrus, a novel automated measure of breast cancer risk based on features of a mammogram, which has an OPERA of 1.7.^28^ A similar OPERA applies to a polygenic risk score based on approximately 3,000 single nucleotide polymorphic markers.^32^ We have also found that defining breast density at higher pixel brightness thresholds can give stronger risk prediction,^33^, ^34^, ^35^, ^36^ and that familial risk could be stronger if based on a continuous measure using multigenerational pedigree data.^37^ It remains to be seen how much addition of these breast cancer risk factors might improve risk stratification.

In summary, our findings illustrate the potential for improving mammography screening. Women at increased risk of serious breast cancers that occur within regular screening intervals could be more accurately identified than by using BI‐RADS alone. Information routinely collected by some mammography services could be used to better classify women in terms of risk. Triaging women for breast screening might be substantially improved by considering breast density as a continuum, adjusting it for BMI and using other measures related to risk. This would also give women with dense breasts, and their physicians, more information about the consequences of having dense breasts—an increased risk of developing an interval breast cancer, a critically important outcome for screening programs and women.

## Supporting information


**Appendix S1**: Supporting information

## Data Availability

The datasets used for the current study are available upon reasonable request from the corresponding author.
