# Supplementary material for: Interval breast cancer risk associations with breast density, family history and breast tissue aging
Source: Int J Cancer. 2019 Nov 12;147(2):375–82. doi: 10.1002/ijc.32731 (PMC7318124; doi:10.1002/ijc.32731)
Supplement: Supplementary file 1 — Appendix S1: Supporting information [file IJC-147-375-s001.docx]

**Rate of breast tissue ageing**

For fitting this model we used Rosner et al [1] from Nurses’ Health Study estimation for the multiple full-term livebirths modification model (see Table 2 and Figure 4 of Rosner et al [1]). Whereas *n* = number of full-term livebirth, *t* = current age, *t*_0_ = age at menarche, *t*_1_ = age at first full-term livebirth, *t*_2_ = age at last full-term livebirth and *t*_m_ = age at menopause women, d(*t*) is ‘breast tissue ageing’ at sequential age *t*: *d_1_* = 0.21 (the decrease in the rate of ‘breast tissue ageing’ after first full-term livebirth), *d_2_* = 0.03 (the decrease in rate of ‘breast tissue ageing’ for each full-term livebirth after the first full-term livebirth), *d_3_* = 0.26 (the decrease in rate of ‘breast tissue ageing’ at menopause), *k* is determined by the rate of increasing in breast cancer incidence with ‘breast tissue ageing’: *k_1_* = 7.61 (the increase in cumulative of ‘breast tissue ageing’ at first full-term livebirth), *k_2_*= –1.69 (the increase in cumulative of ‘breast tissue ageing’ at second full-term livebirth) and *k_3_* = 0.68 (the increase in cumulative of ‘breast tissue ageing’ at menopause). We assumed the number of years interval for each full-term livebirth after the first full-term livebirth were the same and we averaged them: $\left( \frac{t_{2} - t_{1}}{n -1} \right)$.

Consequently, we had six sub-models to calculate the rate of ‘breast tissue ageing’, whereas for sub model 1, we considered women were pre-menopause and had no parous:

*d(t)* = *t* – *t_0_*

For sub model 2, we considered women were post-menopause and had no parous:

*d(t)* = *t_m_* + (*t* – *t_m_*) (1– *d_3_*) – *k_3_* – *t_0_*

For sub model 3, we considered women were pre-menopause and had one full-term livebirth:

*d(t)* = *t_1_* + (*t* – *t_1_*) (1– *d_1_*) + *k_1_* – *t_0_*

For sub model 4, we considered women were post-menopause and had one full-term livebirth:

*d(t)* = *t_1_* + (*t_m_* – *t_1_*) (1– *d_1_*) + (*t* – *t_m_*) (1– *d_1_* – *d_3_*) + *k_1_* – *k_3_* – *t_0_*

For sub model 5, we considered women were pre-menopause and had multiple full-term livebirth:

*d(t)* = *t_1_* + $\left. \left( \frac{t_{2} - t_{1}}{n -1} \right) \right.\left( 1- d_{1} \right)\left( n-1 \right)$ – $\left( \frac{t_{2} - t_{1}}{n -1} \right)d_{2}\left( \frac{\left( n-1 \right)\left( n-2 \right)}{2} \right)$ + (*t* – *t_2_*) (1– *d_1_* – (*n*-1) *d_2_*) + *k_1_* + *k_2_* – *t_0_*

And lastly for sub model 6, we considered women were post-menopause and had multiple full-term livebirth:

*d(t)* = *t_1_* + $\left. \left( \frac{t_{2} - t_{1}}{n -1} \right) \right.\left( 1 - d_{1} \right)\left( n-1 \right)$ – $\left( \frac{t_{2} - t_{1}}{n -1} \right)d_{2}\left( \frac{\left( n - 1 \right)\left( n - 2 \right)}{2} \right)$

+ (*t_m_* – *t_2_*) (1 – *d_1_* – ($n-1$) *d_2_*) + (*t* – *t_m_*) (1– *d_1_* – (*n*-1) *d_2_* – *d_3_*) + *k_1_* + *k_2_* – *k_3_* – *t_0_*

**References**

1. Rosner B, Colditz GA, Willett WC. Reproductive risk factors in a prospective study of breast cancer: the Nurses' Health Study. Am J Epidemiol 1994;139(8):819-35.
